# Supplementary material for: Human parasagittal dura is a potential neuroimmune interface
Source: Commun Biol. 2023 Mar 11;6:260. doi: 10.1038/s42003-023-04634-3 (PMC10008553; doi:10.1038/s42003-023-04634-3)
Supplement: Supplementary file 2 — Supplementary Information [file 42003_2023_4634_MOESM2_ESM.pdf]

Supplementary File

Human parasagittal dura is a potential neuroimmune interface

Erik Melin[1,2], Geir Ringstad[3,4], Lars Magnus Valnes[5], Per Kristian Eide[2,5,*]

[1]Dept. of Radiology, Østfold Hospital Trust, Grålum, Norway, [2]Institute of Clinical Medicine, Faculty of Medicine, University of Oslo, Oslo, Norway, [3]Dept. of Radiology, Oslo University Hospital - Rikshospitalet, Oslo, Norway, [4]Dept. of Geriatrics and Internal medicine, Sorlandet Hospital, Arendal, Norway, [5]Dept. of Neurosurgery, Oslo University Hospital-Rikshospitalet, Oslo, Norway

***Corresponding author:**
Email: p.k.eide@medisin.uio.no (PKE)

Supplementary Table 1. Patient material.

	Demographic				Diagnosis categories						
PatID	Age	Male	Female	BMI (kg/m^2)	Reference	Arach cyst (AC)	Pineal cyst (PC)	IIH	SIH	Hydrocephalus (HC)	Dementias (D)
**80**	69		1	26,6						1	
**82**	46		1	35,0			1				
**105**	34		1	24,2	1						
**108**	39		1	27,4			1				
**109**	44		1	26,8			1				
**111**	20		1	32,3			1				
**122**	74	1		26,3						1	
**124**	45	1		27,1		1					
**125**	39		1	27,8	1						
**126**	47	1		25,9	1						
**128**	32	1		24,9	1						
**129**	47		1	24,6	1						
**133**	44	1		30,1					1		
**135**	69		1	32,7						1	
**137**	44		1	34,1	1						
**145**	38		1	28,3		1					
**146**	37		1	32,6	1						
**160**	36		1	26,8	1						
**161**	23	1		24,3	1						
**162**	32	1		30,7			1				
**167**	46		1	29,4			1				
**171**	24	1		33,1						1	
**172**	29		1	23,8				1			
**173**	44	1		27,5	1						
**174**	47		1	24,7			1				
**175**	25		1	31,1	1						
**176**	29		1	23,3			1				
**178**	26		1		1						
**180**	32		1	20,7		1					
**182**	38		1	30,1			1				
**183**	34		1	17,6					1		
**184**	48	1		24,7		1					
**185**	72		1	20,3					1		
**187**	30		1	30,5			1				
**188**	58	1		25,1		1					
**189**	20		1	30,9		1					
**190**	56		1	32,5					1		
**191**	30		1	23,4	1						
**192**	54		1	29,7					1		
**193**	52	1		26,5					1		
**195**	35		1	24,2		1					
**197**	55		1	34,3							1
**198**	23		1	24,1	1						
**199**	28		1	24,1	1						
**202**	75	1		24,8		1					
**205**	40		1	34,9				1			
**207**	27	1		18,6						1	
**208**	29		1	33,5				1			
**210**	40		1	35,2				1			
**211**	51	1		32,4							1
**214**	19		1	34,4				1			
**215**	28		1	28,4	1						
**218**	37		1	35,4	1						
**219**	38	1		23,0				1			
**220**	70		1	27,1		1					
**222**	45	1		31,1		1					
**223**	71		1	32,3						1	
**225**	50	1		26,0	1						
**226**	56	1		32,4						1	
**227**	32		1	28,7	1						
**228**	60	1		27,2						1	
**229**	69		1	21,8			1				
**230**	55		1	27,7				1			
**235**	35		1	22,4	1						
**236**	35		1	30,1				1			
**237**	22		1	25,4				1			
**239**	25		1	32,0				1			
**240**	30		1	40,8				1			
**242**	40	1		32,6					1		
**244**	41		1		1						
**245**	71		1	23,0	1						
**246**	55		1	32,8	1						
**248**	53		1	31,7						1	
**249**	63		1	20,8					1		
**250**	52	1		27,6						1	
**252**	26		1	35,3				1			
		**22**	**54**		**23**	**10**	**11**	**12**	**8**	**10**	**2**

Demographic data of the patient material. BMI: Body mass index. IIH: Idiopathic intracranial hypertension. SIH: Spontaneous intracranial hypotension. In two subjects BMI was not recorded.

Supplementary Table 2. Volume measures.

	Volume measures				
PatID	PSD (mm³)	GM (L)	WM (L)	CSF (L)	ICV (L)
80	5838			0,5097	1,4827
82	3445	0,5205	0,4793	0,2905	1,4275
105	4197	0,5412	0,4527	0,2225	1,4066
108	3636	0,5067	0,3973	0,2413	1,2985
109	5325	0,5864	0,5204	0,2567	1,5519
111	3618	0,5209	0,3904	0,1452	1,2376
122	4381	0,5963	0,4376	0,5345	1,7082
124	5683	0,5804	0,4925	0,3199	1,6274
125	3822	0,5235	0,4081	0,1842	1,4322
126	5079	0,6088	0,5046	0,32962	1,6774
128	4706	0,5787	0,4239	0,2707	1,4507
129	4980	0,5802	0,5249	0,248	1,5366
133	4605	0,5766	0,5450	0,2729	1,6135
135	1221	0,5362	0,4014	0,3752	1,5611
137	3492	0,5309	0,4272	0,3205	1,4281
145	2192	0,5441	0,4787	0,3447	1,5777
146	4204	0,5478	0,5041	0,2385	1,4503
160	6114	0,5501	0,4319	0,2545	1,4134
161	9654	0,6485	0,4689	0,3049	1,5781
162	5475	0,6012	0,5062	0,203	1,5028
167	5158	0,5185	0,4324	0,288	1,3930
171	8307	0,6778	0,6154	0,6776	2,2392
172	1495	0,5647	0,4681	0,1463	1,3435
173	6023	0,6956	0,6553	0,2719	1,8375
174	4980	0,5039	0,4526	0,1548	1,2854
175	3599	0,6154	0,4095	0,210022	1,4315
176	4410	0,5196	0,4073	0,2051	1,2877
178	3996	0,4879	0,3300	0,2009	1,1793
180	5886	0,5955	0,4532	0,21	1,3857
182	3389	0,5025	0,3613	0,243	1,2669
183	3618	0,5365	0,4324	0,1932	1,2831
184	5480	0,5982	0,4871	0,21563	1,5457
185	7914	0,5014	0,4054	0,2847	1,3236
187	3600	0,5513	0,4346	0,1954	1,3321
188	7097	0,5385	0,4178	0,23422	1,4089
189	4970	0,6124	0,4012	0,1749	1,3638
190	5889	0,5366	0,4207	0,2626	1,3509
191	2599	0,6178	0,4541	0,2274	1,4704
192	3016			0,2564	1,2306
193	6769	0,5552	0,4814	0,3267	1,5712
195	2843	0,5383	0,4686	0,1393	1,4707
197	3736	0,5719	0,5130	0,3736	1,5886
198	2207	0,5597	0,3993	0,202	1,3124
199	3632	0,5813	0,4249	0,1752	1,2837
202	4552	0,5012	0,3918	0,3791	1,3892
205	4107	0,5230	0,4868	0,1959	1,4216
207	3582	0,5709	0,4201	0,3221	1,4224
208	2516	0,5486	0,4468	0,2002	1,3411
210	1762	0,5182	0,4144	0,2056	1,2946
211	3924	0,5034	0,4580	0,3665	1,4326
214	1814	0,5654	0,4398	0,1673	1,3719
215	4463	0,5786	0,4798	0,2709	1,5087
218	3737	0,5702	0,4811	0,2304	1,4583
219	1718	0,4442	0,3752	0,2581	1,2368
220	9051	0,5394	0,4214	0,3527	1,4441
222	9554	0,5978	0,5039	0,2209	1,6048
223	7998	0,4508	0,3563	0,4284	1,3744
225	847			0,402	1,5401
226	1704			0,9391	1,7442
227	1554	0,5957	0,4756	0,2115	1,4428
228	5867	0,5552	0,4369	0,4962	1,6689
229	6000	0,5068	0,4413	0,3358	1,4436
230	3079	0,5321	0,4715	0,2182	1,3538
235	2919	0,4861	0,4405	0,1784	1,2816
236	588	0,5287	0,4391	0,1554	1,3306
237	1219	0,5078	0,3912	0,1479	1,2093
239	4870	0,5397	0,4513	0,2844	1,4589
240	1097	0,5914	0,4656	0,2061	1,4419
242	4445	0,6383	0,5671	0,2766	1,7358
244	4108	0,5496	0,4787	0,3291	1,5065
245	1656	0,4821	0,3783	0,2906	1,3017
246	3563	0,5268	0,4582	0,3633	1,5238
248	1052	0,5707	0,4584	0,422	1,5271
249	5163	0,5180	0,4108	0,2622	1,3711
250	6508			1,1159	2,1766
252	1024	0,5976	0,4282	0,16	1,3700

PSD: Parasagittal dura. GM: Gray matter. WM: White matter. CSF: Cerebrospinal fluid. ICV: Intracranial volume. In five subjects, volumes of GM and WM were not computed due to image quality.

Supplementary Table 3. Peak levels in PSD and blood.

PatID	MRI: Time to max enrichment	Tmax (plasma)
**137**	24,0	14,1
**175**	6,0	4,6
**176**	24,0	8,7
**178**	48,0	5,1
**182**	24,0	7,9
**183**	6,0	11,0
**184**	48,0	6,6
**187**	24,0	8,1
**188**	48,0	7,0
**189**	24,0	8,5
**192**	24,0	10,2
**193**	24,0	14,7
**195**	6,0	5,1
**198**	48,0	4,1
**199**	6,0	4,5
**202**	24,0	9,2
**207**	48,0	11,9
**208**	6,0	4,1
**210**	24,0	6,0
**215**	6,0	4,4

In a subset of patients, time (hours) to peak tracer enrichment in PSD at MRI and time (hours) to peak plasma concentrations were compared. MRI: Magnetic resonance imaging. T_{max}: Time to maximum plasma concentration.

Supplementary Table 4. Static and pulsatile ICP scores versus tracer enrichment in PSD at 24 hours.

	PSD: Percentage Change compared with Pre	Overnight ICP	
PatID	24 hr	Average Mean ICP	Average MWA
175	28,6	0,8	3,0
182	420,4	8,7	2,1
189	242,5	8,6	3,1
193	291,7	4,6	4
199	29,7	2,7	3,3
202	375,6	11	4,2
205	-23,5	18,3	10,3
207	35,6	8,3	4,1
208	60,0	12,1	6,6
210	11,0	13,9	9,3
211	257,1	3,8	2,9
215	-20,0	11,4	3,9
218	248,0	8,7	4,4
219	25,3	0,2	4,7
223	280,2	5,6	4,1
227	263,4	9,6	3,8
228	54,3	6,2	3,7
230	1,0	7,7	5,0
236	8,4	12,6	6,9
239	13,0		5,4
248	33,6	9,8	5,1
250	16,9		4,3
252	30,4	16,7	9,5

In a subset of patients, the overnight average of static ICP (average mean ICP) and pulsatile ICP (average MWA) were compared with percentage change in tracer enrichment in PSD (i.e. percentage change in normalized T1 signal). PSD: Parasagittal dura. MWA: Mean wave amplitude. In two subjects, mean ICP was not recorded.

Supplementary Table 5. Time-dependent tracer enrichment in CSF, PSD and brain.

	CSF				PSD				Cerebral Cortex				Subcortical white matter			
	Percentage Change compared with Pre				Percentage Change compared with Pre				Percentage Change compared with Pre				Percentage Change compared with Pre			
**PatID**	**3 hr**	**6 hr**	**24 hr**	**48 hr**	**3 hr**	**6 hr**	**24 hr**	**48 hr**	**3 hr**	**6 hr**	**24 hr**	**48 hr**	**3 hr**	**6 hr**	**24 hr**	**48 hr**
**80**																
**82**									127,74	182,24	117,67	61,17	8,89	22,48	47,42	35,69
**105**									95,37	130,03	93,07	32,14	3,87	11,48	36,06	19,72
**108**									63,23	120,71	210,06		2,00	6,88	67,58	
**109**									18,63	31,17	23,71	5,90	-2,94	2,01	10,53	-0,68
**111**									115,66	141,16	96,74	46,73	9,21	17,65	34,80	22,10
**122**									21,35	30,97	78,10	56,23	1,01	3,47	28,58	28,41
**124**									32,94	54,99	83,98	41,85	2,98	7,44	31,46	23,15
**125**									33,04	56,45	45,82	22,35	1,70	1,99	17,25	13,52
**126**									4,85	14,86	69,02	51,26	1,57	-0,17	16,27	22,79
**128**									75,68	108,00	102,24	37,65	2,44	8,87	33,80	21,18
**129**									16,99	81,10	53,87	21,67	-3,55	7,24	17,38	7,24
**133**									28,83	52,72	63,50	21,45	0,40	7,58	18,99	11,49
**135**										13,07	82,36			4,04	39,57	
**137**	83,3	239,9	739,5	263,3	83,5	216,1	649,1	221,3	52,82	79,13	135,74	48,84	-0,11	4,99	47,71	24,84
**145**									33,96	75,03	120,19	74,37	8,12	20,25	56,00	56,17
**146**									140,84	168,45	126,01	43,96	4,85	12,12	37,27	18,27
**160**									57,64	109,17	111,91	55,98	2,80	13,03	43,33	30,72
**161**									12,32	29,64	43,40	30,24	3,08	0,90	20,55	16,33
**162**									19,03		103,21				31,60	
**167**									104,86	139,70	178,50	91,08	12,02	18,26	70,36	50,04
**171**									52,81	93,37	183,11	101,95	3,06	5,76	47,06	41,64
**172**									109,77	150,56	111,49	38,46	3,81	14,04	44,93	21,77
**173**									24,61	44,24	70,53	26,68	3,13	1,55	23,94	14,08
**174**									79,37	113,22	76,57	35,56	5,80	13,46	31,26	18,97
**175**					212,2	300,7	28,6	-11,7	36,29	63,04	47,63	22,87	-6,15	-2,58	21,57	9,12
**176**			572,6	213,8			371,4	124,7	172,68	188,31	127,47	56,90	14,06	20,00	52,49	34,38
**178**	33,0	49,3		60,9	-22,2	-5,1		25,8	41,31	71,45	52,64	29,12	1,36	4,74	22,62	16,10
**180**	150,9	433,3	282,4	177,4	15,1	247,2	219,9	106,3	98,25	127,67	109,91	48,03	6,43	11,87	36,15	22,83
**182**	98,4	593,3	615,1	302,8	32,7	310,1	420,4	121,6	110,36	149,35	129,80	58,57	10,98	13,86	47,40	30,21
**183**	181,3	481,9	436,0	204,0	86,8	243,0	229,8	139,1		107,98	120,14	47,00	1,62	7,53	43,69	28,77
**184**	-1,3		170,4	125,0	-5,2		37,3	116,8	30,15	45,44	42,19	28,12	1,58	2,56	12,33	11,24
**185**									12,84	25,71	32,02	12,76	-2,45	2,64	10,04	3,88
**187**	174,5	340,3	297,9	128,5	15,7	84,9	272,1	-21,4	90,88	166,81	122,48	57,99	11,84	19,94	45,04	34,23
**188**	-6,9	34,2	168,5	170,8	-6,9	27,5	44,0	106,3	5,02	16,14	48,98	33,88	0,37	1,08	17,00	17,71
**189**	424,3	483,7	378,4	223,9	176,7	186,8	242,5	127,5	59,65	102,03	77,59	37,59	7,58	9,90	36,99	25,83
**190**									33,27	59,01		35,40	2,52	4,64		16,36
**191**									60,43	88,79	62,23	35,51	-2,37	4,73	19,50	12,90
**192**		264,0	367,1	166,0		176,5	291,0	112,5								
**193**	7,0	27,9	395,9	376,0	0,9	12,5	291,7	234,1	20,90	58,75	110,31	61,10	-0,93	-3,15	30,76	27,99
**195**	75,8	387,6	238,4	95,2	56,7	187,4	119,6	85,1	14,00	41,32	32,00	16,25	-1,38	3,42	11,18	10,42
**197**	60,2	333,7	460,2	437,2	23,1	101,3	178,9	187,5	21,97	109,80	127,64	61,84	-0,94	10,98	39,72	29,34
**198**	15,7	100,5	96,5	107,6	12,2	25,7	14,0	43,7	47,56	82,28	37,24	24,02	-2,16	8,84	11,70	10,89
**199**	41,1	207,0	344,6	222,1	38,1	132,0	29,7	41,2	29,36	94,83	80,29	31,53	-2,67	8,25	26,05	20,04
**202**	6,2	-0,6	421,8	332,8	24,4	12,6	375,6	294,4	2,04	16,34	100,33	87,87	-4,15	-4,42	31,99	41,34
**205**	-16,7	-19,2	-7,2	-15,2	-35,7	-36,1	-23,5	-40,5	35,11	54,98	28,94	9,24	-0,78	3,04	9,41	2,61
**207**	14,5	118,0	407,7	243,9	-7,4	46,2	35,6	87,6			125,44	67,60			37,52	24,22
**208**	495,2	541,5	502,7	334,9	124,8	247,1	60,0	29,7	85,31	131,90	89,17	49,56	1,55	12,82	35,91	26,48
**210**			62,8	63,3			11,0	6,9	55,09		95,99	74,25	2,16		27,47	29,77
**211**		358,8	453,7			134,4	257,1			85,55	156,84	110,28		8,45	43,85	38,56
**214**									59,16	99,99		34,44	6,19		13,88	22,05
**215**	-15,8	-9,9	53,7	96,3	9,3	17,0	-20,0	16,9	25,20	46,69	27,94	14,48	-2,13	2,10	7,75	3,25
**218**	450,8	491,9	394,8	167,6	211,0	271,2	248,0	160,5	54,26	105,63	96,44	42,14	1,06	8,58	33,35	20,42
**219**	-25,2	-17,7	23,3	13,9	-10,5	6,6	25,3	-14,7	11,38	31,50	34,85	25,37	2,18	-3,57	10,77	14,16
**220**	9,4	-8,3	123,2	146,4	4,5	-6,6	43,8	132,8	64,33	107,65	97,23	64,15	4,56	12,07	37,68	31,65
**222**									30,99	74,76	57,67	29,99	-1,69	6,06	20,79	15,00
**223**	17,8	68,2	373,8	466,8	31,8	-2,1	280,2	311,8	53,52	92,13	117,82	81,56	2,32	13,29	45,63	36,22
**225**	10,2	0,4	127,1	135,3	9,6	0,4	59,0	72,5								
**226**																
**227**	465,2		433,5	165,3	169,8		263,4	188,5	43,13	114,98	105,40	51,67	-1,86	15,22	31,32	28,20
**228**	22,4	32,4	127,4	178,3	9,0	12,9	54,3	157,8	21,15	44,16	93,57	65,87	4,04	6,56	33,24	36,23
**229**									23,30	34,58	13,57	9,46	0,25	2,40	1,00	4,33
**230**	104,7	335,0	210,1	221,1	104,7	259,2	1,0	22,9	31,80	106,29	82,52	46,88	1,90	9,39	30,09	22,00
**235**	99,6	313,5	356,1	177,6	18,8	169,2	159,7	94,2	30,58	84,06	77,74	30,70	-4,45	5,33	24,01	15,64
**236**	3,0	8,3	74,0	97,4	20,6	17,1	8,4	12,7	23,87	69,18	75,30	48,89	-5,74	3,38	18,40	11,62
**237**	343,7	454,8	561,7	522,4					64,72	123,16	166,58	77,39	5,22	12,15	57,10	38,78
**239**	-8,7	42,2	90,8		5,2	30,3	13,0		44,67	85,42	71,91		3,47	9,23	25,93	
**240**									35,88	54,25	67,89	35,93	5,42	5,86	19,32	20,12
**242**	20,2	19,2	52,2	28,0	16,1	5,9	29,8	17,0	9,29	29,74	31,94	16,16	-2,29	-1,53	11,74	8,88
**244**	8,8	35,1	308,6	301,3	4,4	21,2	59,5	49,6	16,31	56,43	60,41	33,85	-2,19	5,25	19,99	18,51
**245**	-10,7	-19,6	66,4	112,9	-5,5	-13,1	-24,1	5,5	22,38	32,16	68,48	50,83	1,51	-0,39	18,80	20,68
**246**	52,4	418,6	480,7	242,4	31,9	112,0	308,4	220,6	31,30	92,84	111,43	52,93	-1,19	7,75	36,73	25,58
**248**	8,7	-16,1	11,1	75,4	-2,7	-24,9	33,6	57,6	2,10	8,37	38,11	27,76	1,87	1,12	10,44	12,06
**249**	-2,2	3,1	192,5	270,0	1,2	4,1	154,0	139,8	22,32	54,54	95,58	64,01	-0,77	8,42	29,76	32,61
**250**		16,9	40,7	24,9		1,5	16,9	-2,6								
**252**	725,2		270,8	170,6	75,9		30,4	70,9	44,52	105,10	83,72	48,29	-7,44	12,17	27,48	24,58

The time-dependent change in tracer enrichment (measured as percentage change in normalized T1 signal units) in different compartments, including CSF space, PSD, cerebral cortex and subcortical white matter. Missing data were due to lacking MRI sequences, images not useful for FreeSurfer segmentation, or other technical issues with images.
