# Supplementary material for: Human parasagittal dura is a potential neuroimmune interface
Source: Commun Biol. 2023 Mar 11;6:260. doi: 10.1038/s42003-023-04634-3 (PMC10008553; doi:10.1038/s42003-023-04634-3)
Supplement: Supplementary file 3 — Reporting Summary [file 42003_2023_4634_MOESM3_ESM.pdf]

nature portfolio

Corresponding author(s): Per Kristian Eide

Last updated by author(s): Jan 18, 2023

Reporting Summary

Nature Portfolio wishes to improve the reproducibility of the work that we publish. This form provides structure for consistency and transparency in reporting. For further information on Nature Portfolio policies, see our Editorial Policies and the Editorial Policy Checklist.

Statistics

For all statistical analyses, confirm that the following items are present in the figure legend, table legend, main text, or Methods section.

n/a	Confirmed	
☐	☒	The exact sample size (*n*) for each experimental group/condition, given as a discrete number and unit of measurement
☐	☒	A statement on whether measurements were taken from distinct samples or whether the same sample was measured repeatedly
☐	☒	The statistical test(s) used AND whether they are one- or two-sided<br>*Only common tests should be described solely by name; describe more complex techniques in the Methods section.*
☐	☒	A description of all covariates tested
☐	☒	A description of any assumptions or corrections, such as tests of normality and adjustment for multiple comparisons
☐	☒	A full description of the statistical parameters including central tendency (e.g. means) or other basic estimates (e.g. regression coefficient) AND variation (e.g. standard deviation) or associated estimates of uncertainty (e.g. confidence intervals)
☐	☒	For null hypothesis testing, the test statistic (e.g. *F*, *t*, *r*) with confidence intervals, effect sizes, degrees of freedom and *P* value noted<br>*Give P values as exact values whenever suitable.*
☒	☐	For Bayesian analysis, information on the choice of priors and Markov chain Monte Carlo settings
☒	☐	For hierarchical and complex designs, identification of the appropriate level for tests and full reporting of outcomes
☐	☒	Estimates of effect sizes (e.g. Cohen's *d*, Pearson's *r*), indicating how they were calculated

Our web collection on statistics for biologists contains articles on many of the points above.

Software and code

Policy information about availability of computer code

Data collection	The MRI-data was collected from the hospital picture archiving and communication system (PACS); Sectra IDS7 (Sectra, Sweden)
Data analysis	Image analysis: For assessment of PSD volumes 3D FLAIR images were used. First, PSD in eight patients were segmented manually in ITK-SNAP, version 3.8.0, after thresholding in Slicer, version 4.11.20200930. The eight PSD segmentations were used to train an in-house AI-software. The AI-model segmented PSD in another seven patients; These segmentations were reviewed and corrected in ITK-SNAP and used as training data for the AI-software. The remaining 61 patients were segmented by the AI-model and controlled slice per slice manually. The AI-model was used for efficient segmentation; The accuracy of the model was not estimated and all segmentations from the AI-model were reviewed and corrected manually if needed. For each patient, T1-GRE images from all time points were aligned to a subject-specific template using FreeSurfer, version 6.0, to assess signal change in the brain after contrast. For five patients the registration failed. Total intracranial volumes were calculated from T1-GRE with SPM12. For all time points in 46 patients, ROIs were manually placed in PSD and adjacent CSF on T1-BB images in the hospital picture and archiving system (PACS), Sectra version 7 (Sectra AB, Sweden) for signal intensity measurements in PSD and adjacent CSF.<br><br>Statistics: The statistical analysis was performed using SPSS version 27 (IBM Corporation, Armonk, NY).

For manuscripts utilizing custom algorithms or software that are central to the research but not yet described in published literature, software must be made available to editors and reviewers. We strongly encourage code deposition in a community repository (e.g. GitHub). See the Nature Portfolio guidelines for submitting code & software for further information.

Data

Policy information about availability of data

All manuscripts must include a data availability statement. This statement should provide the following information, where applicable:

- Accession codes, unique identifiers, or web links for publicly available datasets
- A description of any restrictions on data availability
- For clinical datasets or third party data, please ensure that the statement adheres to our policy

The authors have access to all data used in this study. Anonomized data may be provided on request.

Human research participants

Policy information about studies involving human research participants and Sex and Gender in Research.

Reporting on sex and gender	We have divided male/female based on biological attribute and used the term sex throughout the manuscript. Sex was one of the variables studied.
Population characteristics	76 patients were investigated, 54 female and 22 male. Age: 42.6±15.0. All subjects were under work-up for CSF disorder: Arachnoid cysts; Pineal cysts; Idiopathic intracranial hypertension; Spontaneous intracranial hypotension and hydrocephalus conditions. After evaluation at the clinic some patients, with no clear CSF disorder, were classified in the reference group or in the dementia group.
Recruitment	Patients referred to the neurosurgical department at Oslo University Hospital for tentative CSF disorder were included.
Ethics oversight	The study was approved by the Institutional Review Board (2015/1868), the Regional Ethics Committee (2015/96) and the National Medicines Agency (15/04932-7). The patients were included after written and oral informed consent.

Note that full information on the approval of the study protocol must also be provided in the manuscript.

Field-specific reporting

Please select the one below that is the best fit for your research. If you are not sure, read the appropriate sections before making your selection.

☒ Life sciences ☐ Behavioural & social sciences ☐ Ecological, evolutionary & environmental sciences

For a reference copy of the document with all sections, see nature.com/documents/nr-reporting-summary-flat.pdf

Life sciences study design

All studies must disclose on these points even when the disclosure is negative.

Sample size	No sample-size calculations were performed. The data and correlations between different disease categories, PSD volumes and CSF dynamics presented in this study have never been presented before and the results of this study were difficult to predict. We included all available patients, but the number of patients investigated with intrathecal contrast are limited.
Data exclusions	Eighty-two patients who underwent intrathecal contrast-enhanced MRI were examined. Of these, six patients had to be excluded after imaging due to difficulties to segment PSD for various reasons: three patients with diffuse dural thickening, two with poor image quality from motion artefacts, and one with cerebral venous sinus thrombosis. Exclusion of some images based on poor image quality was expected.
Replication	All images were obtained with the same MRI scanner, same coil and same imaging parameters. The images were analysed with the same software and parameters and finally manually controlled to ensure comparable results within this study. The aim was to measure PSD volumes and correlate them to other variables. The volumes per se are not comparable to previous PSD volume studies where other MRI sequences were used.
Randomization	This was an observational study with no randomization.
Blinding	The clinical diagnosis were not known to the investigator who performed the image analysis

Reporting for specific materials, systems and methods

We require information from authors about some types of materials, experimental systems and methods used in many studies. Here, indicate whether each material, system or method listed is relevant to your study. If you are not sure if a list item applies to your research, read the appropriate section before selecting a response.

Materials & experimental systems

n/a	Involved in the study
☒	☐ Antibodies
☒	☐ Eukaryotic cell lines
☒	☐ Palaeontology and archaeology
☒	☐ Animals and other organisms
☐	☒ Clinical data
☒	☐ Dual use research of concern

Methods

n/a	Involved in the study
☒	☐ ChIP-seq
☒	☐ Flow cytometry
☐	☒ MRI-based neuroimaging

Clinical data

Policy information about clinical studies

All manuscripts should comply with the ICMJE guidelines for publication of clinical research and a completed CONSORT checklist must be included with all submissions.

Clinical trial registration: This was an observational study and therefore not registered in ClinicalTrials.gov.

Study protocol: The study is registered in Oslo University Hospital Research Registry: ePhorte 2015/1868.

Data collection: The study included patients under work-up for tentative CSF disorder at Oslo University Hospital. FLAIR, T1-GRE and T1-BB images and intracranial pressure (ICP) measurements were part of the investigation. Time study period was November 2016 to December 2019.

Outcomes: Predefined primary outcome measure: Volume of parasagittal dura measured on FLAIR images; secondary outcome measure: Signal intensity after intrathecal contrast injection measured on T1-GRE and T1-BB images, intracranial volumes measured on T1-GRE, ICP measured with an ICP sensor in the brain, sleep quality assessed by questionnaire and concentration of tracer in plasma from blood samples.

Magnetic resonance imaging

Experimental design

Design type: Structural MRI, not functional MRI

Design specifications: No functional MRI data to specify

Behavioral performance measures: No functional MRI data to address

Acquisition

Imaging type(s): Structural MRI images: T1-GRE, FLAIR and T1-BB

Field strength: 3 Tesla

Sequence & imaging parameters: All sequences were obtained in the sagittal plane with isotropic 1 mm voxels. T1-GRE: repetition time (TR) = shortest (typically 5.1 ms); echo time (TE) = shortest (typically 2.3 ms); echo train length (ETL) = 232; flip angle = 8°; 1 average
T1-BB: TR = 700 ms; TE = 35 ms; ETL = 55; flip angle = 80°; 2 averages
FLAIR: TR = 4800 ms; TE = 311 ms; inversion time = 1650 ms; ETL = 167; flip angle = 90°; 2 averages

Area of acquisition: Whole brain and cranium.

Diffusion MRI: ☐ Used ☒ Not used

Preprocessing

Preprocessing software: The only preprocessing performed was z-normalization of the signal values before input to the AI-software

Normalization: The signal in the T1-GRE and T1 -BB images were normalized to orbital fat and ocular bulb to correct for change in overall signal intensity after contrast injection. The T1-GRE images for each subject were linear aligned to an subject-specific template for assessment of signal intensity variations over time.

Normalization template: An subject-specific average template was used for each subject.

Noise and artifact removal: none used

Volume censoring: not performed

Statistical modeling & inference

Model type and settings	This was an anatomical and physiological study exploring the volumes and contrast dynamics in the PSD, not brain function.
Effect(s) tested	No tasks were performed

Specify type of analysis: ☐ Whole brain ☐ ROI-based ☒ Both

Anatomical location(s)	Whole brain analysis were performed to estimate intracranial volumes and contrast dynamics in the brain. ROI-based analysis were used to measure PSD volumes and contrast dynamics in PSD and adjacent CSF.
Statistic type for inference (See Eklund et al. 2016)	Not relevant; No fMRI data were analysed
Correction	Not relevant; No fMRI data were analysed

Models & analysis

n/a	Involved in the study
☒	☐ Functional and/or effective connectivity
☒	☐ Graph analysis
☒	☐ Multivariate modeling or predictive analysis
